# Interplay between Learning and Voluntary Wheel Running in Male C57BL/6NCrl Mice

**DOI:** 10.3390/ijms24054259

**Published:** 2023-02-21

**Authors:** Laura Niiranen, Ville Stenbäck, Mikko Tulppo, Karl-Heinz Herzig, Kari A. Mäkelä

**Affiliations:** 1 Research Unit of Biomedicine and Internal Medicine, Faculty of Medicine, University of Oulu, 90220 Oulu, Finland; 2 Pediatric Gastroenterology and Metabolic Diseases, Pediatric Institute, Poznan University of Medical Sciences, 60572 Poznan, Poland; 3 Medical Research Center (MRC), Oulu University Hospital, 90220 Oulu, Finland

**Keywords:** voluntary exercise, wheel running, cognition, learning, memory, IntelliCage, metabolism, PhenoMaster

## Abstract

Exercise is shown to improve cognitive function in various human and animal studies. Laboratory mice are often used as a model to study the effects of physical activity and running wheels provide a voluntary and non-stressful form of exercise. The aim of the study was to analyze whether the cognitive state of a mouse is related to its wheel-running behavior. Twenty-two male C57BL/6NCrl mice (9.5 weeks old) were used in the study. The cognitive function of group-housed mice (n = 5–6/group) was first analyzed in the IntelliCage system followed by individual phenotyping with the PhenoMaster with access to a voluntary running wheel. The mice were divided into three groups according to their running wheel activity: low, average, and high runners. The learning trials in the IntelliCage showed that the high-runner mice exhibited a higher error rate at the beginning of learning trials but improved their outcome and learning performance more compared to the other groups. The high-runner mice ate more compared to the other groups in the PhenoMaster analyses. There were no differences in the corticosterone levels between the groups, indicating similar stress responses. Our results demonstrate that high-runner mice exhibit enhanced learning capabilities prior to access to voluntary running wheels. In addition, our results also show that individual mice react differently when introduced to running wheels, which should be taken into consideration when choosing animals for voluntary endurance exercise studies.

## 1. Introduction

Exercise is known to have beneficial effects on cognition and learning, and it has been widely studied in animal models and humans [1,2,3,4,5,6,7,8]. Cognition itself consists of various main domains such as sensation, perception, motor skills, attention, memory, and language functions [9]. Impairments and decline of cognitive functions are associated with adverse health effects [10,11,12]. While the effects of exercise on cognitive functions are well studied, the correlation of cognitive function to the preference for voluntary exercise is unclear.

Laboratory mice are often used as a model to study the effects of exercise and cognition in preclinical studies [1,2,13]. Different aspects of behavior and cognitive functions can be studied in mice [14], e.g., depression-related behavior with forced swim test [15], anxiety with open maze and elevated zero maze tests [16], and cognitive functions with Barnes and Y maze tests [17]. The IntelliCage system offers the platform to study spontaneous behavior patterns, spatial and temporal learning, social and hierarchical behaviors, and conditioned behavior as well as discrimination learning and preferences with various preprogrammed trials with minimal human interference in an enriched and stress-free environment [18,19,20]. The PhenoMaster system provides information on metabolism with indirect calorimetry and behaviors, such as food and water intake, home cage activity, exploratory activity, and the system can also be equipped with a running wheel to monitor voluntary exercise [21].

Running wheels are generally used in research to act as an exercise model in laboratory mice [22], investigating several different research questions, such as the adaptive response of cardiac and skeletal muscle to exercise [23] and the effects of exercise on tumor growth in a humanized mouse model [24] and on cognitive functions [25,26]. Compared to treadmills, running wheels provide a voluntary form of exercise and mice run spontaneously for extended periods of time and distances [27]. Laboratory mice use the running wheels readily, but also mice in the wild are known to frequently use them when provided [28]. Wild mice have been observed to use running wheels with food reward, but also in the absence of reward, indicating willingness to run voluntarily. Due to the voluntary nature of the exercise, it has been suggested that using a wheel running is non-stressful for the animals [22,29]. However, it should be noted that exercise itself alters the behavior of the mice, and the wheel running behavior can be affected by several different confounding factors created by the genetic background and environment [30]. Wheel running time, velocity, and distance [31] can fluctuate depending on the strain [32], age [33], and sex [32,33] of the mice. Environmental and physiological factors, such as the availability of food [30] and stress [34], play an important role in the locomotor activity of laboratory mice. Short fasting bouts can increase locomotor activity, whereas fasting of six hours or more reduces physical activity, which is linked to a torpor-like behavior (reduced metabolic state) [35]. Stress is known to increase wheel running activity compared to non-stressed mice [34], which might be a form of stereotypic behavior to alleviate stress [34,36,37,38].

Mice have been selectively bred for increased voluntary wheel running behavior [39,40]; however, the majority of the studies use common laboratory mouse strains to model endurance training without screening the strains or individual animals for their physical activity preference [41]. Our previous observations suggest that individual mice have great variation in their voluntary running wheel activity and willingness to initiate and maintain wheel running. Here we studied whether there is an association between an innate cognitive function with voluntary wheel-running activity. The mice were first evaluated for their learning capabilities with specialized behavioral trials (place learning and reverse learning) in the IntelliCage system [18,42,43], followed by behavioral and metabolic monitoring in the PhenoMaster with access to the voluntary running wheels.

## 2. Results

**Figure 1:**
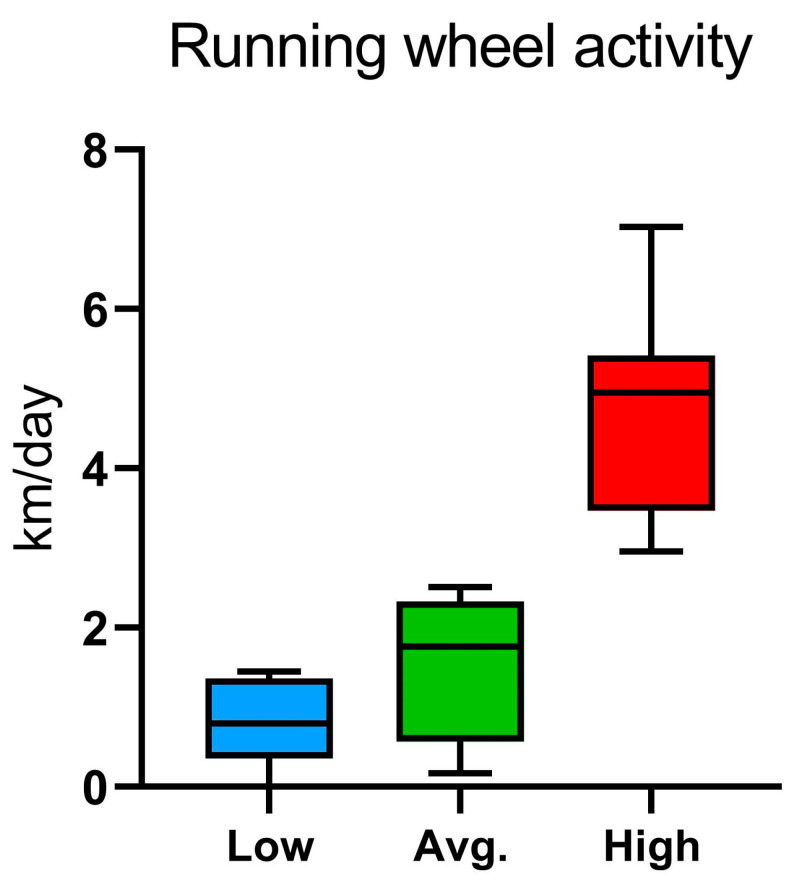
Study groups assigned according to the running wheel activity in PhenoMaster, categorized as kilometers (km) per day into low runners (Low; n = 7), average runners (Avg.; n = 8), and high runners (High; n = 7). Data are presented as mean and standard deviation.

A total of the 22 male C57BL/6NCrl mice underwent first the cognitive testing protocol (17 days) in the IntelliCage and then were individually phenotyped (17 days) in the PhenoMaster system. To analyze the results of the learning trials from the IntelliCage and behavioral parameters from the PhenoMaster, the mice were divided into three groups: low runners (Low; n = 7), average runners (Avg.; n = 8), and high runners (High; n = 7) according to their running wheel activity (running distance as km/day) measured in the PhenoMaster (Figure 1).

### 2.1. Learning and Cognitive Function: IntelliCage Results

**Figure 2:**
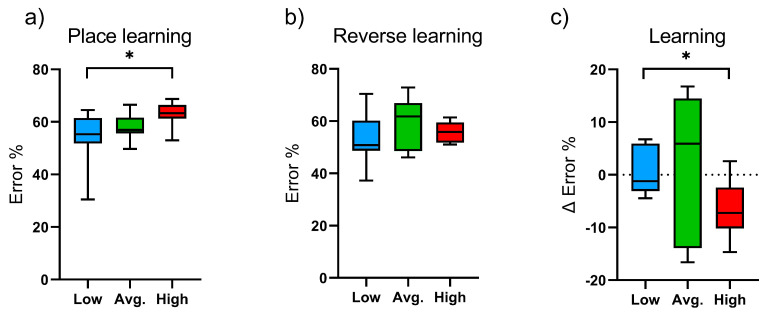
Learning of the running wheel groups (Low; n = 7, Avg.; n = 8, High; n = 7) in the IntelliCage. Error percentages in the (**a**) place learning and (**b**) reverse learning trials and the (**c**) relative learning. Statistically significant differences are indicated with an asterisk: * *p* < 0.1. Data are presented as mean and standard deviation.

There were no differences between the three groups in the number of visits or nose pokes in the IntelliCage operant corners during the free adaptation. The number of licks per hour differed significantly between low and average runners (*p* = 0.019) during free adaptation, with increased licks in low runners. No difference was observed in the above-mentioned parameters during the nose-poke adaptation trial between the groups. High-runner mice had a higher error percentage than low-runner mice in the place learning trial (*p* = 0.053). No difference was detected between the groups in errors in the reverse place learning trial, however, high runners showed relatively fewer errors in the reverse place learning task compared with low runners (*p* = 0.035) indicating improved learning performance as the learning trials progressed (Figure 2).

### 2.2. Voluntary Wheel Running, Home-Cage Activity, and Metabolic Parameters: PhenoMaster Results

The running distances of the three running wheel groups differed (Low; 0.81 ± 0.58 km, Avg.; 2.05 ± 0.43 km, High; 4.77 ± 1.33 km) with most of the running activity occurring during the dark hours (Table S1). Even though the three groups of mice differed significantly according to their wheel running activity, there were no differences in the home-cage activity in the PhenoMaster. It should be noted that the groups exhibited no differences in rearing activity in the PhenoMaster, which is a form of exploratory behavior. Most of the mice utilized the running wheels readily except four individuals with either low or excessive running wheel behavior. Two animals in the low-runner group displayed low or delayed onset in the running wheel activity whereas two of the high-runner group mice exhibited excessive running, lowered food intake, and hypometabolism (torpor-like behavior).

**Figure 3:**
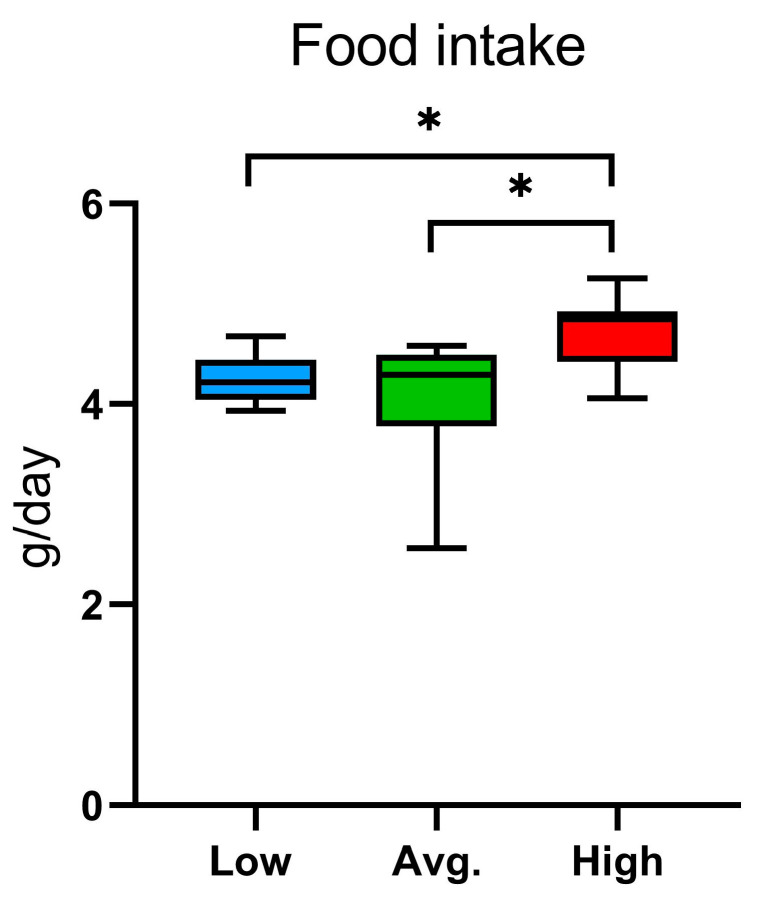
Food intake in the running wheel groups (Low; n = 7, Avg.; n = 8, High; n = 7) in the PhenoMaster. Statistically significant differences are indicated with an asterisk: * *p* < 0.1. Data are presented as mean and standard deviation.

The high runners had significantly higher food intake compared with both the average (*p* = 0.020) and the low runners (*p* = 0.028) (Figure 3). However, the drinking behavior did not differ between the groups in the PhenoMaster. No difference was observed in the respiratory exchange ratio (RER) between the groups (Table S1).

### 2.3. Body Weights and Urine Corticosterone

**Figure 4:**
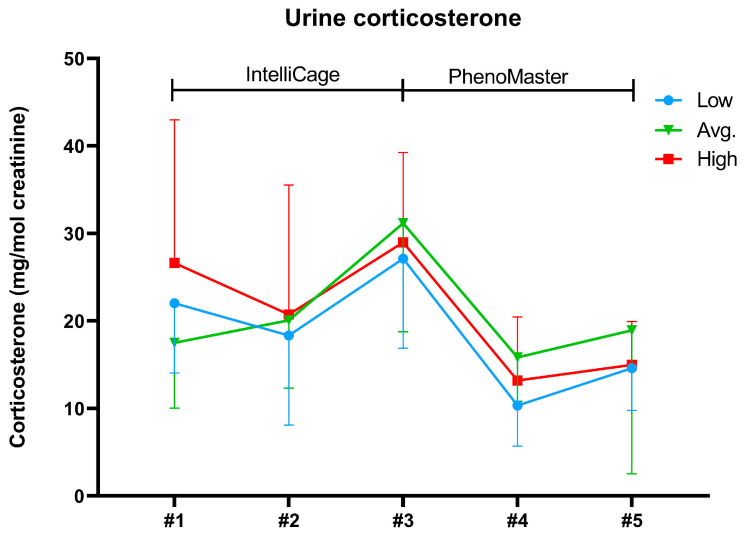
Urine corticosterone levels normalized to creatinine in the running wheel groups (Low; n = 7, Avg.; n = 8, High; n = 7) in sample collection points #1–#5. Data are presented as mean and standard deviation.

**Figure 5:**
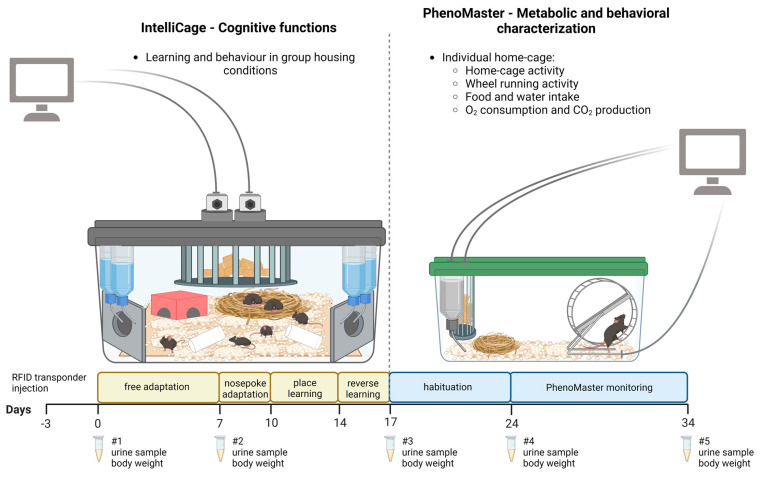
Simplified schematic representation of the study design including the IntelliCage and PhenoMaster measurements. RFID (radiofrequency identification) transponders were injected subcutaneously into 9.5-week-old mice at the beginning of the study. Urine samples and weights were collected at five time points throughout the experimental setup (#1–#5). Created with Biorender.com.

The body weights of the three groups of mice did not differ during the study (Table S1). There was no difference in urine corticosterone levels between the groups. However, the deviations in corticosterone levels were relatively large inside some groups and time points. The urine corticosterone followed a similar pattern in all three groups and was increased in sample #3 after the learning trials (place learning, reverse learning), whereas in samples #4 and #5 the levels were lower when the animals were in single housing conditions in the PhenoMaster (Figure 4 and Figure 5).

## 3. Discussion

Wild-type laboratory mice, not selectively bred for high voluntary wheel running, are frequently used when studying endurance training [41]. In the present study, we investigated the interplay between cognitive function and susceptibility to voluntary wheel running exercise in young adult male C57BL/6NCrl mice. After the experimental protocol (34 days: 17 days in IntelliCage and 17 days in PhenoMaster), voluntary running activity was measured, and the mice were categorized into three different groups (low, average, and high runners). High runners exhibited enhanced learning abilities over low runners albeit no significant differences were observed with average runners. Additionally, high runners had significantly higher food intake compared with average and low runners to compensate for the increased energy expenditure. In general, C57BL/6 mice run from approximately 2 km/day to over 15 km/day on running wheels with females running longer distances than males [23,32,33,44,45,46]. In the present study, mice exhibited variation in the wheel running behavior in groups as well as individually. In low runners, some of the mice initiated wheel running after a delay or otherwise exhibited extremely low wheel running activity, whereas two of the high runner mice exhibited excessive running behavior.

In our study, high runners had more errors in the first learning trial (place learning) but improved their performance as the learning trial progressed to the second test (reverse learning) compared with low runners. This indicates improvement in learning but might also suggest impulsive behavior, which could contribute to their higher preference for voluntary wheel running. Previous studies on the correlation of cognition and wheel running activity in mice have shown variable outcomes. House mice selected for high voluntary wheel running had enlarged midbrains with no change in hippocampal, cerebellar, and forebrain volumes when compared with control mice [47]. Rhodes and colleagues studied spatial learning in female Hsd:ICR mice that were selected for high voluntary wheel running [39,48]. The authors found that mice with high running activity did not improve their spatial learning in the Morris water maze test after 40 days of access to the running wheels compared to control mice. However, high runners showed high neurogenesis in the hippocampus and an addiction to exercise, which resembles a feature of human ADHD [49]. Thus, a high preference for voluntary wheel running may indicate traits of addictive or hyperactive behavior. 

Wheel running behavior could in addition to the addictive implications represent a type of stereotypic behavior in mice [28,34,36,37,38]. Recently, Mason and Würbel suggested that wheel running may model exercise addiction rather than normal exercise behavior [38]. Stereotypic striped mice used running wheels to redirect their stereotypic behavior [37]. In addition, wheel running seems to fulfill the criteria for stereotypic behavior in mice [38]. In our study, two mice exhibited excessive running behavior.These individuals did not show increases in their urine corticosterone, but the excessive wheel-running behavior likely represented stereotypic behavior, which should also be taken into consideration when using laboratory mice for exercise studies. 

In addition to the excessive running behavior, some mice expressed a lack of or markedly reduced physical activity. Furthermore, few mice initiated wheel running after a delay and one animal displayed only two short wheel running bouts. Binder and colleagues reported that 12–14 weeks old male C57BL6/N mice initiated wheel running within only a couple of days after the introduction to the running wheels [46]. On the other hand, Bartling and colleagues showed different levels of running wheel activities between individual mice [33]. However, despite the apparent differences in the running distances between the animals, male mice ran during the first week and even increased their performance for the next measurement point. Similar fluctuation in wheel running activity has been reported in C57BL/6 mice [22]. In that study, one mouse seemed to stop running completely on day 14 and then initiated running again on the following day. In the same study, mice had a burst in their running wheel activity during days 3 or 4. An increase in voluntary wheel running may affect other activity behavior, and high or low wheel running can be reflected as compensatory behavior in other physical activity patterns in the home cage. In our study, the locomotor activity measured via the infrared beams surrounded by the home cages was similar in low runners compared with average and high runners, indicating that all groups had similar baseline activity. Additionally, rearing activity, in which the mouse stands on the hind legs when exploring the environment, was similar in the three groups in the PhenoMaster measurement.

Our mice had similar stress responses since there was no significant difference in the urine corticosterone levels between the running wheel groups. Urine corticosterone levels exhibited similar changes between the sampling time points between the groups. All three groups had the highest level of urine corticosterone after the place learning and reverse learning trials, suggesting that the learning trials increased their stress level. The corticosterone levels decreased in all three groups after the mice were transferred to the individual measurements in the PhenoMaster system. Male mice may have higher stress levels in group housing conditions compared with single housing as they are more susceptible to aggressive behavior in a group hierarchy [50,51]. As the learning trials in the IntelliCage were designed to analyze learning and behavior in a group setting, female mice were preferentially used over male mice [18]. Here, we wanted to study the learning and preference for voluntary exercise in male mice, as they have been generally more commonly used in scientific research and exercise studies.

The strengths of our study include the study setting, in which the learning and memory functions are tested prior to access to voluntary running wheels. Studying the learning and behavior in male mice is also a strength, as male mice have been generally more commonly used in exercise studies than female mice. As a weakness of our study, the effective study group size was relatively small due to practical limitations in the equipment for group size and measurement repetitions (IntelliCage and PhenoMaster). 

## 4. Materials and Methods

A total of 34 healthy male C57BL/6NCrl mice were investigated in the study, of which a total of 22 mice underwent the complete study in IntelliCage and PhenoMaster. Mice were bred in a mouse barrier unit and maintained in groups of 5–6 until they were transferred to the research facilities for the IntelliCage investigations at the age of 9.5 weeks. Mice had ad libitum access to chow (Teklad Global Rodent diet T.2018C.12, Harlan Teklad, USA) and water unless otherwise mentioned and kept under standard environmental conditions throughout the study with the temperature at 21 ± 2 °C, relative humidity at app. 40% and illumination from 6 a.m. to 6 p.m. with one hour increasing and decreasing dim light periods. The mice were housed in groups since weaning to reduce the likelihood of aggressive behavior until the individual PhenoMaster measurements.

Mice first underwent the learning trials in the IntelliCage in groups, followed by individual metabolic and behavioral assessment in the PhenoMaster system with access to the running wheels (Figure 5).

### 4.1. IntelliCage Measurements

IntelliCage (NewBehavior, TSE IntelliCage Plus SW, TSE Systems, Berlin, Germany) is an automated home-cage system for the measurement of cognitive functions in an enriched environment with social group interaction. The system consists of a large home cage (55 × 37.5 × 20.5 cm) with four computer-operated operant corners and up to 16 mice can be recorded simultaneously. The environment includes narrow tunnels and hiding places and enables nose-poking behavior to study the new environment. IntelliCage utilizes transponders for the identification (RFID: radio frequency identification) of individual mice during the measurement. The operant corners are accessed through a short tunnel that contains sensors that detect the presence of the mice via temperature and identify the individuals’ transponder, which is recorded as a “visit”. There is a small chamber at the end of the tunnel with two round doors that open to enable access to the two drinking bottles and the drinking is recorded as the number of “licks”. Access to the water bottle is usually coupled with a successful “nose poke” to the door in the different preprogrammed learning events as positive reinforcement. A short air puff is used as negative reinforcement to discourage lingering in the operant corners. The system records the number of all visits to the corners, nose pokes, and licks from the bottle, which can be used to determine the learning behavior and cognitive function of a mouse. 

The identifying transponders for the mice were implanted subcutaneously to the back of the neck under isoflurane anesthesia the same day the mice were transferred to the research facilities from the barrier unit. After the implantation, mice were allowed to recover for three days before the initiation of the IntelliCage measurements. The cage was equipped with aspen bedding (Tapvei^®^, Paekna, Estonia) and nesting material as well as four red transparent tunnels placed in the middle of the cage. Mice had ad libitum access to food throughout the study and water access according to the IntelliCage learning trials. If the animal stayed in the operant corner longer than 5 min, an air puff was delivered to encourage the mouse to leave the corner. 

Our IntelliCage measurement/workflow consisted of free adaptation, nose-poke adaptation, place learning, and reverse place learning events (Figure 5). The free adaptation period lasted for seven days during which all doors were open and mice had free access to the water bottles. During the free adaptation period, the exploratory behavior and circadian activity of the mice were monitored. In the three-day nose-poke adaptation, the doors were closed and the mouse needed to learn to poke the door in order to open it and access the water. The door closed after 30 s, regardless of the time spent drinking. During the next four days, each individual mouse was permitted to access only one of the previously determined least preferred corners (place learning). This was followed by three days of reversal of the place learning, in which the correct corner access was reversed to the opposite one in the IntelliCage. In place learning and reverse learning, each mouse gained access to the water bottle by nose-poking the correct assigned corner, and the correct and incorrect nose pokes were recorded.

The experiments had to be discontinued for two sets (2 × 6 male C57BL/6NCrl mice) of the mice during IntelliCage measurements due to aggressive behavior and fighting (data not included). These mice were then euthanized. The disruption in the behavior and group dynamics may have been affected by possible noises and vibrations from a nearby construction site. One of the mice in group 2 (average runners) had their microchip replaced during the nose-poke adaptation. Two mice were removed from the study during the PhenoMaster measurements (i.e., running wheel measurements), as they ceased eating leading to torpor. 

### 4.2. PhenoMaster Monitoring and Voluntary Wheel Running 

After the IntelliCage measurements, mice were transferred to the PhenoMaster (WB 2000 KHL, TSE Systems GmbH, Berlin, Germany). Mice were separated from their study groups and placed in individual training cages, which were similar to the actual monitoring cages. The cages were provided with aspen bedding and nesting material and with wooden enrichment blocks (Tapvei^®^, Paekna, Estonia). The water bottle included a lever that the mouse needed to press to get access to drinking water, which the mice learn easily. The monitoring room temperature was kept at 21 ± 1 °C and the relative humidity at 40%. The room lights were on from 6 a.m. to 6 p.m. with increasing and decreasing dim light periods similar to as described for the IntelliCage conditions.

After the training period, mice were measured in individual monitoring cages for 10 days. The PhenoMaster measured food and water intake, home-cage activity (total and rearing activity), and breath gases. The PhenoMaster system enables monitoring and observing the mice in a home-cage environment with minimal human interference. The cages were also equipped with running wheels. The wheels are made of stainless steel and the wheel diameter is 11.5 cm and the width is 4.0 cm. Wheels were freely available for the mice during the monitoring period and checked during the cleaning of the cage to verify the functionality of the running wheels. Cages were changed to clean ones every 7th day. At the same time, the water and food baskets were cleaned, and new water and food were added.

### 4.3. Body Weights and Urine Collection for Corticosterone and Creatinine Analysis 

Body weights were measured and urine was collected before and after the IntelliCage adaptation period, at the end of the IntelliCage measurements (after the reversal training), and before and after LabMaster monitoring (Figure 5). The urine samples were collected at 7 am with a noninvasive method, in which the mouse was placed in an empty clean cage and the urine samples were collected by pipette immediately and stored in −70 °C until further analysis. Urine corticosterone levels were analyzed with ELISA kits according to the manufacturer’s instructions (Corticosterone Parameter Assay Kit, R&D Systems^®^, Minneapolis, MN, USA). Urine creatinine levels were measured with QuantiChrom™ Creatinine Assay kit (BioAssay Systems, Hayward, California, United States) according to the manufacturer protocol. Urine creatinine levels were used to correct the urine corticosterone concentration for differences in kidney function such as glomerular filtration and hydration. The mice were euthanized by cervical dislocation after the PhenoMaster study.

### 4.4. Statistical Analysis

All IntelliCage and PhenoMaster values were divided with the actual time spent in the experiment since minor one-hour differences existed between the experiments. IntelliCage values were divided into hours and PhenoMaster values into days. 

Normality was tested using the Kolmogorov–Smirnov test and normality was not observed. Due to the distribution of the data and relatively small sample size, a non-parametric approach was chosen. Data were grouped using the wheel running activity (distance as km) in a metabolic cage. Grouping was done by dividing the population into three equal-sized groups. The Kruskall–Wallis test was used to study differences between all groups and the Mann–Whitney test to study differences between two individual groups. Due to the small sample size, a *p*-value smaller than 0.10 was chosen as the level of statistical significance. Data were first retrieved from IntelliCage using the NewBehaviorTSE IntelliCage Plus SW, Project 5331, software version 01/2016 and from the PhenoMaster using TSE LabMaster, version 5.7.6, TSE Systems GmbH software in Excel format. Further processing, statistical analysis, and graph generation were done using the IBM SPSS Statistics v.27 and GraphPad Prism (version 9.3.1 for Windows, GraphPad Software, San Diego, CA, USA). All figures are presented as mean and standard deviation.

## 5. Conclusions

C57BL/6NCrl mice exhibit highly variable wheel-running activity between individual animals. This would need to be taken into consideration when designing exercise studies with laboratory mouse models. Mice with the highest wheel running behavior showed enhanced learning capabilities prior to the voluntary exercise screening and higher food intake. However, the enhanced learning features also relatively higher error rate in the first learning trial, which could suggest impulsive traits.

## Data Availability

Not applicable.

## Supplementary Materials

The following supporting information can be downloaded at: https://www.mdpi.com/article/10.3390/ijms24054259/s1.
